# Attred: Attribute Based Resource Discovery for IoT †

**DOI:** 10.3390/s21144721

**Published:** 2021-07-10

**Authors:** Mohammed B. M. Kamel, Yuping Yan, Peter Ligeti, Christoph Reich

**Affiliations:** 1 Faculty of Informatics, Eotvos Lorand University, 1053 Budapest, Hungary; yupingyan@inf.elte.hu (Y.Y.); ligetipeter@inf.elte.hu (P.L.); 2 Institute of Data Science, Cloud Computing and IT Security, Furtwangen University of Applied Science, 78120 Furtwangen, Germany; christoph.reich@hs-furtwangen.de; 3 Department of Computer Science, University of Kufa, Najaf 540011, Iraq

**Keywords:** IoT, resource discovery, ABE, RDHT

## Abstract

While the number of devices connected together as the Internet of Things (IoT) is growing, the demand for an efficient and secure model of resource discovery in IoT is increasing. An efficient resource discovery model distributes the registration and discovery workload among many nodes and allow the resources to be discovered based on their attributes. In most cases this discovery ability should be restricted to a number of clients based on their attributes, otherwise, any client in the system can discover any registered resource. In a binary discovery policy, any client with the shared secret key can discover and decrypt the address data of a registered resource regardless of the attributes of the client. In this paper we propose Attred, a decentralized resource discovery model using the Region-based Distributed Hash Table (RDHT) that allows secure and location-aware discovery of the resources in IoT network. Using Attribute Based Encryption (ABE) and based on predefined discovery policies by the resources, Attred allows clients only by their inherent attributes, to discover the resources in the network. Attred distributes the workload of key generations and resource registration and reduces the risk of central authority management. In addition, some of the heavy computations in our proposed model can be securely distributed using secret sharing that allows a more efficient resource registration, without affecting the required security properties. The performance analysis results showed that the distributed computation can significantly reduce the computation cost while maintaining the functionality. The performance and security analysis results also showed that our model can efficiently provide the required security properties of discovery correctness, soundness, resource privacy and client privacy.

## 1. Introduction

With the continuous upgrading of Internet of Things (IoT) industry in the recent years, the IoT nodes in the perception layer are growing rapidly. It is estimated that by 2025, the number of connected IoT devices reach over 30 billion [1]. There is a wide variety of perception layer nodes, which can be a relatively high degree of intelligence and computation power such as laptops and cell phones, or a relatively low degree of intelligence and computation power such as sensors, cameras, and so on. As the number of IoT nodes grows, the process of discovering them in the IoT network becomes more challenging. Different IoT resources in the perception layer such as sensors have different access control and discoverability requirements. This diversity in the access control requirements, the degree of intelligence and the ability to perform heavy computations, add a number of challenges in the development of an efficient, yet secure resource discovery model. Thus, it is necessary that while setting effective access control mechanisms, reduce the computation cost of the resource discovery models in order to improve the system efficiency.

There are number of researches [2,3,4] that proposed resource discovery models with a centralized entity. The centralized entity in such models has a high computation power that can be used for registration and discovery, but this entity in the discovery model can present a single point of failure and attack. Considering the security, efficiency and privacy issues in the models with a centralized managing entity, various researches replaced the centralized entity with a number of distributed entities utilizing Distributed Hash Table (DHT) [5,6,7,8,9,10]. These models provide domain based discovery of resources in the system. Some researches proposed discovery models based on other schemes such as context based discovery [11] that provide more accurate outputs. However, due to the considerable lower latency of DHT based solutions, DHT is still the underlying used technology in a number of resource discovery models. DHT and using a hash function assigns random identifiers to the nodes. As a result, the nodes are positioned uniformly at random in the overlay, regardless of their physical locations. This prevents DHT based discovery models from considering the physical locations of the nodes during the creation of the overlays. Region-based DHT (RDHT) takes into consideration the physical locations of underlying nodes during overlay generating and node positioning, that lead to more efficient processes in the generated overlay.

Attribute Based Encryption (ABE) can well address the security issues of IoT perception layer. ABE has its advantages of fine-grained access control, one to many mode and privacy-friendly encryption. Users in ABE can be identified by some of their attributes, and the encrypted information can only revealed to authorized users based on their attributes. In an ABE based scheme, both ciphertext and key are related to a set of attributes. According to the characteristics of information and the attributes of clients, the encryptor can customize an encryption strategy, and the generated ciphertext can be decrypted only by the clients whose attribute satisfies the encryption policy. In this way, the encryption mode is converted from one to one to one to many, which is well-suited for distributed IoT environment. Using CP-ABE (Ciphertext-Policy Attribute-Based Encryption), access trees can be defined and be used in the process of encrypting data. On the other hand, the secret keys to decrypt the encrypted data are generated over a set of attributes and any encrypted data can be accessed by having the relevant secret key. As a result, under the encryption scheme of CP-ABE, the data owner is able to encrypt the data and send them publicly, while preventing unauthorized entities from accessing the publicly transmitted data.

In resource discovery, the resources have to be discovered in the network depending on their attributes. In addition to performance requirements [12] such as location-based and multi-attribute discovery, the security of the registered resources has to be guaranteed. Without a proper discovery access control, these resources are vulnerable to being discovered by unauthorized entities. The most important security issues in the resource discovery can be summarized in three main points: access control, privacy and availability. In access control, the address data that are provided by the resources need to be accessible by authorized entities in the network. On the other hand, it is a must that for unauthorized clients, they are forbidden to get access to the address data of the registered IoT resources. The privacy of the entities in the network has to be guaranteed. The issuer of a discovery process provides some information about itself such as its attributes and the attributes of the required resources during the discovery process. This information has to be kept private and only accessible by the trusted entities. Lastly, in the networks that organize the discovery process via a centralized entity, the availability of the system can be attacked, to prevent the authorized entities to discover the required resources. It is essential to maintain the availability of system and guarantee the discoverability of the resources by legitimate and authorities entities.

In order to handle all the obstacles, we proposed Attred, a decentralized model for resource discovery of IoT resources, utilizing RDHT and decentralized ABE (DABE). RDHT as a general overlay for fog computing environment has been modified and implemented in our resource discovery model to construct the fog/edge computing and distribute the registration of the IoT resources. DABE is utilized to ensure that the address data of IoT resources are disocoverable only by the authorized clients, without relying on a centralized entity or a need to direct communication between nodes. Considering the heavy computation requirement in DABE and the resource constrained IoT devices, as part of Attred, we have proposed a secure distributed computation of parts of DABE that require heavy computation without revealing any sensitive data or a real-time cooperation between nodes

The main contributions of our paper are the following:

1. Location-aware discoverability: Decentralized resource discovery using the proposed region based DHT overlay with a proposed tuple data structure that creates a location-aware overlay (considering the physical location of underlying nodes) and allows multi-attribute and location-based resource discovery.
2. Decentralized discovery control: Fine-grained discovery control for clients to discover the address data of the resources based on their attributes without a centralized entity, which fits the distributed nature of IoT environment.
3. Distributed Computation: A secure distributed computation to distribute some of the heavy computations in Attred using additive secret sharing without affecting the security of the model.

The rest of paper is organized as follows. In the second section, we will discuss the related works in resource discovery. The preliminaries will be introduced in the third section for a further understanding. The fourth section explains our modelling and solution. The fifth section includes the evaluation and discussion of our proposal. Finally, our conclusions are stated in last section.

## 2. Related Works

Some researchers adopt the use of a centralized entity as part of their proposed resource discovery models that manages some parts or all parts of the system. Authors in [2] have proposed a large scale resource discovery to discover the devices and sensors in the IoT network by building a scalable architecture called Digcovery. The framework enables the users to register their resources into a shared infrastructure and to access/discover accessible resources by a mobile phone. Their proposed work focuses on the discoverability of devices based on context-awareness and geo-location. Digcovery allows high scalability for the discovery based on a flexible architecture. The proposed model relies on a centralized point called digcoverycore for management and discovery. Jia et al. [3] proposed a discovery model for IoT that performs the discovery based on various constraint parameters. A centralized directory server in this model registers the services and can be used to discovery the registered services by the clients. The discovery is done using semantic service description method OWL-S${}^{iot}$ that describes both the IoT services and discovery requests. Cheshire and Krochmal [4] proposed a Domain Name System (DNS) based discovery for the IoT network. It defines a model on how the users register their resources and discover the resources based on the DNS protocol. The proposed model does not modify the underlying DNS protocol messages and codes and as a result is simple to implement. In this model, a centralized authority stores the registered resources and there is no additional security consideration to the original DNS protocol itself. Using the centralized scheme helps organizing the resources in an entity that has a high computation capability, however, this centralized entity might turn into a single point of failure, which, if fails, the overall system stops. This profoundly affect the availability and reliability of the system. Additionally, the centralized entity could turn into a bottleneck for the system affecting the overall system performance.

IoT networks that rely on a centralized entity such as cloud suffer from several disadvantages [13]. With billions of connected devices that have to transfer data to the cloud issues such as efficiency, privacy and security will be raised. In IoT applications that have to communicate with the cloud which is on the other edge of the network a significant amount of traffic has to be used for this process, which could affect the overall efficiency of the system. In addition, for access control, the cloud server should interact with each user to define the access scope and privilege. The system efficiency will be highly affected as the number of users increases. Because of a huge number of devices in the IoT network, this scheme cannot achieve the scalability. In contrast, fog computing [14] extends the cloud computing to the edge of the network (i.e., close to the point of origin of the data). Processing the data locally helps to achieve scalability, and at the same time will mitigate the potential risks against privacy and security breach of data.

Several researches utilized the distributed scheme in the IoT network as a method for resource discovery. The authors in [5] proposed a single-gateway based hierarchical DHT solution (SG-HDHT) for an efficient resource discovery in Grids (i.e., Virtual Organizations (VO)). The model defines a global DHT and number of second level DHTs, a DHT overlay for each VO. A single super peer node in a second level DHT overlay is attached to the global level DHT. The resource discovery request in the proposed model is directed to the super peer of the VO and then through the global DHT to the super peer of the requested resource. Paganelli et al. [6] proposed a layered architectural design that identifies three main features: multi-attribute indexing, range query support and peer-to-peer routing. The design approach is based on the selection of an over-DHT indexing scheme to design a three layered functional architecture. Space Filling Curve (SFC) [15] layer that is used for multi-dimensional to one-dimensional mapping, Prefix Hash Tree (PHT) [16] layer that is used to leverage the generic DHT interfaces and a DHT layer that is implemented based on Kademlia [17].

Authors in [7] proposed an architecture consists of two discovery levels, local and global service discovery. It uses the P2P scheme for resource discovery, and IoT gateways are the peers in the P2P overlay. This model supports location aware discovery and uses two layers: the Distributed Location Service (DLS) as a DHT based architecture that provides required information to access any resource in the network depending on its URL and the Distributed Geographic Table (DGT) [18] that distributes the information depending on the location of nodes. Tanganelli et al. [9] proposed a fog based discovery model that consists of two layers. It utilizes DHT to create the two-layered overlay and, nodes in the overlay are divided into master and normal nodes. An inner global DHT layer includes the master nodes, and there are a number of clusters that represents the second layer in the overlay. The global DHT is created using a hash function, and the clusters are generated using locality preserving hash function. While the overlay in this model is created without considering the physical location of nodes, the location based registration and discovery can be done through the two clusters of latitude and longitude. Cabrera et al. in [11] proposed a discovery model for smart cities. Unlike location/domain based models such as DHT based models, their proposed model uses urban context to spread service description in different urban places. The services are stored in locations that are more likely to be discovered based on a similarity parameter. This model provides a more precise and higher rate of resolved outputs comparing to location and domain based models, but it comes at the cost of higher overhead and delay. In addition, its scalability needs to be further discussed and explored.

Although the discussed models support important features such as location-aware, context based and multi-attribute discovery, but the required security and privacy restrictions and considerations in the process of resource registration and discovery need to be further studied. Authors in [8] proposed a model of resource discovery in IoT. The proposed model uses DHT to register the resources in IoT network. It allows the resources to be discovered based on their attributes. In addition, some resources can be registered privately and be discovered only by a predefined set of users. This is done by encrypting the address data of the resources using symmetric encryption (i.e., AES) prior to registering them into public data lake. The authors in [19] proposed a two layer resource discovery that uses different methods with symmetric encryption to create a private layer of discoverable resources. However, these models are based on a two level binary policy and cannot define the set of attributes of clients that are able to discover the registered resources. In other words, the clients either can discover a private resource (by having the symmetric key) or they are not able to get the addresses of private resources. Pahl and Liebald [20] introduced a distributed modular directory of service properties and a query federation mechanism based on virtual state layer (VSL) [21]. This model supports multi-attributes as well as adding new attributes in real-time. The model addresses some security requirements such as role-based access model.

ABE can greatly enriches the flexibility of encryption strategy and user authority in fog computing, and expands from the one-to-one mode to one-to-many mode. It can effectively achieve non-interactive and decentralized access control and makes the authentication and access control to be done without any need to a centralized trusted third party. Guo et al. [22] proposed a model to distribute the sensitive data of patients by adopting a key policy ABE scheme. Their model includes multi attribute authorities that define different organizations. While the attribute authorities are responsible for key generation, the actual data are stored in the cloud service providers. Blockchain has been utilized to ensure the integrity and traceability of registered data in the system. Authors in [23] utilized ABE to secure the request of the issuer of the lookup process during the resource discovery. ABE was used to add important features to this proposed model. Security and privacy are considered during the service discovery process by protecting the user’s requests and restricting the access to the discovery of a service. In addition, adopting ABE in the discovery restricts the access to the resources in the system. One of the drawbacks of this model is the possibility of attacking the availability of a service provider by a malicious participant.

Authors in [10] proposed a decentralized resource discovery model that adopts ABE as the encryption scheme for encrypting the addresses of the registered resources in the system. It ensures that the resources can control the required clients by defining the access policy during the registration process. Although the model includes a central authority, the registration process can be done without a direct connection to attribute authorities. Wang et al. [24] proposed a distributed ABE for discovery in mobile social networks. Their proposed model utilizes multi-authority ABE that achieves the fine grained access control and privacy without additional special signatures and the initiator encrypts the information with an access policy defined by itself. In addition to keep the data as close as possible to the registered resources by utilizing RDHT and allowing location aware discovery, Attred aims to allow clients only by their inherent attributes, to discover the resources in the network. Considering the limited computation power of some IoT devices, Attred allows the resources to distribute the workload of resource registration between different nodes in RDHT and removes any centralized entity that might turn into a potential single point of attack and failure. Table 1 shows the supported properties in the studied resource discovery models.

## 3. Preliminaries

In this section, bilinear mapping, security assumptions and some definitions relate with ABE are given. In addition, we state the mechanisms of ABE, DHT and resource discovery in IoT.

### 3.1. Security Definitions

**Definition** **1** (Additive secret sharing). *Let $F_{p}$ be a finite field of order p, $s\in F_{p}$ be a secret, and $P=\{P_{1},P_{2},...P_{n}\}$ be a set of n parties that the secret s has to be shared with. The additive shares of the secret s are n−1 integers that are chosen uniformly at random from $F_{p}$, and represent the first n−1 shares ($s_{1}\overset{R}{\leftarrow }F_{p},s_{2}\overset{R}{\leftarrow }F_{p},\cdots ,s_{n-1}\overset{R}{\leftarrow }F_{p}$). The nth share is computed as $s_{n}=\left(s-\sum_{i=1}^{n-1}s_{i}\right)modp$. A share $s_{i}$ in the set $\left\{s_{1},s_{2},\cdots ,s_{n}\right\}$ is sent to the party $P_{i}$. Given all the shares, the secret s can be constructed as $s=(\sum_{i=1}^{n}s_{i})modp$*.

**Definition** **2** (Access structure). *Let $P=\{P_{1},P_{2},...P_{n}\}$ be a set of n parties. An access structure is a collection* Γ *of non-empty subsets of P, i.e., $\Gamma \subseteq 2^{\left\{P_{1},P_{2},...P_{n}\right\}}$. Any U∈Γ is called authorized set, and any X∉Γ is called unauthorized set. We say* Γ *is monotonic access structure if for any M,N, if M∈Γ and M⊆N, then N∈Γ.*

**Definition** **3** (Bilinear pairing). *Let G be an additive group of points of an elliptic curve over a finite field with prime order p and $G_{1}$ be multiplicative group of prime order p, and let g∈G be a generator. A pairing is a map $e:G\times G\to G_{1}$, which satisfies the following properties:*

*a*. *Non-degeneracy: ∃g∈G,e(g,g)≠1;*
*b*. *Bilinearity: $\forall x,y\in Z_{p},\forall g,h\in G$, $e\left(g^{x},h^{y}\right)=e{\left(g,h\right)}^{xy}$;*
*c*. *Computability: ∀g,h∈G, there exists an efficient algorithm to compute e(g,h).*

Bilinear mapping is a function in which elements in two linear spaces can generate elements in the third linear space, and all parameters in the function are linear. Previously, it has been used in attack models in the elliptic curve cryptography. However, now it plays a more important role in encryption structures, especially in ABE [25]. The security of ABE protocols are related to various hardness assumptions, here we only refer to one [26] which the proposed scheme is based on:

**Definition** **4** (General Subgroup Decision (GSD) assumption). *Let G be a bilinear group of composite order and let $S_{0},S_{1}$ be two distinct subgroups of G. Given a random element from $S_{b}$, it is hard to determine b∈{0,1}, even if given a random element from several subgroups $S_{i}$ that each satisfies $S⋂S_{0}=\emptyset =S⋂S_{1}$ or $S⋂S_{0}\neq \emptyset \neq S⋂S_{1}$.*

### 3.2. Distributed Hash Table

DHT is a distributed storage and lookup system that provides an efficient lookup mechanism in the system. As in hash tables [27], the data are stored in different nodes in DHT as a key/value pair. The value parameter in this pair includes some information about the corresponding key, such as its URL. The stored values in DHT can be retrieved from the overlay based on the key parameter in the key/value pair. Most DHT systems assign a seemingly unique identifier to each of the nodes in the overlay during joining the system. The keys can be generated using collision-resistant one-way hash function. A used hash function in DHT is fed with information about the node such as node IP [28].

**Definition** **5** (collision-resistant one-way hash function). *A function H(.) that maps an arbitrary length input m into a fixed length digest d is called collision-resistant one-way hash function if it satisfies the following properties:*

- *Easy Computation: given m, it is easy to compute H(m).*
- *One-way: given h, it is hard to find any m such that h=H(m). This property is also called preimage resistance.*
- *Strong Collision Resistance: it is hard to find two distinct messages $m^{\prime }\neq m^{\prime \prime }$ with $H\left(m^{\prime }\right)=H\left(m^{\prime \prime }\right)$.*

A newly joined node is positioned in the overlay based on the output of the used hash function, i.e., its identifier. Using identifiers instead of other types of addressing (e.g., IPs) helps to balance the data storage among participating nodes without any centralized entity. In addition to load balancing, it solves the scalability by providing the service of generating the identifiers by the participating nodes themselves. Although context oriented solution [11] provides more accurate lookup result, this accuracy comes at the cost of considerable overhead and delay comparing to DHT based lookup system. There are several protocols to implement DHT such as Chord [28], Kademila [17], Pastry [29] and Tapestry [30]. The lookup result accuracy in DHT lookup can be improved by different ranking algorithms, such as [31]. DHT uses a large address space of integer numbers. The size of the address space depends on the fixed output size of the function that is used to generate the randomize identifier. The size of the key space is same as the address space, i.e., the same function is used to generate identifiers for nodes and keys for the stored pairs. To achieve the uniform distribution of data among all participating nodes the collision-resistant one-way hash function is used in DHT.

DHT has two implementation interfaces: put and get. The put interface takes the key/value pair and stores this pair in the DHT. The get interface takes a single parameter key and lookup the stored pair in the DHT to retrieve the corresponding value to the key. In DHT the store (i.e., put interface) and lookup (i.e., get interface) operations are done with an upper bound of O(log(E)), in which *E* is the number of nodes in the DHT. This feature guarantees that any participating node in DHT can store a pair of key/value or lookup based on given key by routing through of maximum log(E) nodes. One of the main drawbacks of DHT based system in the inconsistency between the physical underlay and the generated DHT overlay. This leads to significant delay during the store and lookup phases. Region-based Distributed Hash Table (RDHT) [32] is a special implementation of DHT that divides the main overlay into several regions and the nodes are positioned in a specific region in the overlay based on their physical locations. RDHT takes into consideration the physical locations of both nodes in the underlay and stored data in the system during overlay generating, node positioning, and store/lookup phases. This feature brings two additional advantages to the system: First, RDHT has lower latency during store/lookup phases comparing to store/lookup phases in DHT, since the values are registered in physically close peers in the overlay. Second, due to location-aware overlay, the lookup phase can be done based on the physical locations of peers.

### 3.3. Resource Discovery in IoT

The IoT resources can be IoT data, IoT service, or IoT objects. Therefore, the search mechanism should be able to find either IoT data, IoT service, IoT object, or a mixture of them. The search techniques can be functional (event-based, location-based, time-related, content-based, spatio temporal-based, context-based, real-time and user interactive searching) or implementational (text-based, metadata-based or ontology-based approach) [33]. The resource discovery is a mechanism to return the address of a resource based on the information provided during the lookup operation. The resource address could be its Uniform Resource Identifier (URI), other metadata, and further links about the resource. Among the first steps of adding a resource (i.e., an IoT thing, a meta-data, or a service provided by an IoT thing) to the Internet of things network is registering of that resource in the IoT network. Later on and depending on the used architecture in the network (i.e., centralized or decentralized), this registered resource is discoverable through a single or multiple points in the network. An essential characteristic of the IoT is the avoidance of single point of failures as it can be a centralized discovery service, even if implemented using redundancy and replication. One of the main goals of a decentralized discovery approach is to keep the data as close as possible to the point of origin. Therefore, avoidance of a single point of failure and save the data close the its origin point are two main features of using a decentralized scheme rather than a centralized one. As the connected devices become more powerful in terms of connectivity and computing power, this goal becomes a realistic and necessary to achieve.

### 3.4. Attribute Based Encryption

In contrast to the traditional public key encryption algorithms, the decryptor in ABE is a subset of users, not a single one which is possible by introducing the concept of attributes. It uses the combinations of subsets’ attributes as the public key to encrypt all the data, while the private key is calculated and assigned to the individual by the attribute authority based on the user attribute. Standing on the bilinear pairing (Definition 3) techniques, the ABE builds the various access structures to achieve fine-grained access control of data.

In 2005, Sahai and Waters [34] first proposed the concept of Fuzzy Identity Based Encryption (FIBE), which leads to the further development on ABE mechanism. In contrast to the traditional Identity-Based Encryption (IBE), the set of attributes is considered as a user’s identity in this scheme. In conventional IBE, the encrypted message and the private keys are generated based on the identified information of the user, thus, only a specific user can decrypt the message. In this case, it is a single one-to-one communication mode, compared to FIBE that works as a one-to-many communication mode.

ABE can be viewed as a generalization of IBE. It provides a new solution for access control of encrypted data, enabling one-to-many communication. The system informatively introduces an access structure in the public encryption, where ciphertext or private keys can be generated according to it, and only users who satisfy the formulated conditions can decrypt the ciphertext. In this system, the user’s private key is created by a central authority (CA). Based on the attributes of a user, the system describes the user with a set of attributes and the CA generates the corresponding private key for the user upon the set of attributes. The ciphertext in ABE is not encrypted for a specific user, but it is generated based on a collection of attributes. On the basis, in order to provide more complex access control polices, two ABE formations have been proposed, which are Key-Policy Attribute-Based Encryption (KP-ABE) [35] and Ciphertext-Policy Attribute-based Encryption (CP-ABE) [36].

Goyal et al. [35] first proposed Key-Policy Attribute-Based Encryption (KP-ABE) scheme. In KP-ABE, the ciphertext is associated with the attribute set and the access structure is embedded in the key. Only when the attribute set satisfies the access structure policy of a key holder, the key holder can decrypt the data successfully. This scheme adopts a monotonic access tree structure, and it allows for “AND”, “OR” and threshold access control operations on attributes.

Cipher-Policy Attribute-Based Encryption (CP-ABE) was first proposed by Bethencourt et al. [36]. In CP-ABE, the private key is associated with the attribute set and the access structure is embedded in the ciphertext. Only when the attribute set satisfies the access structure policy of the data owner, can the user decrypt the ciphertext to get data. It uses a tree access structure, which can achieve the access control operations of “AND” and “OR”.

**Figure 1:**
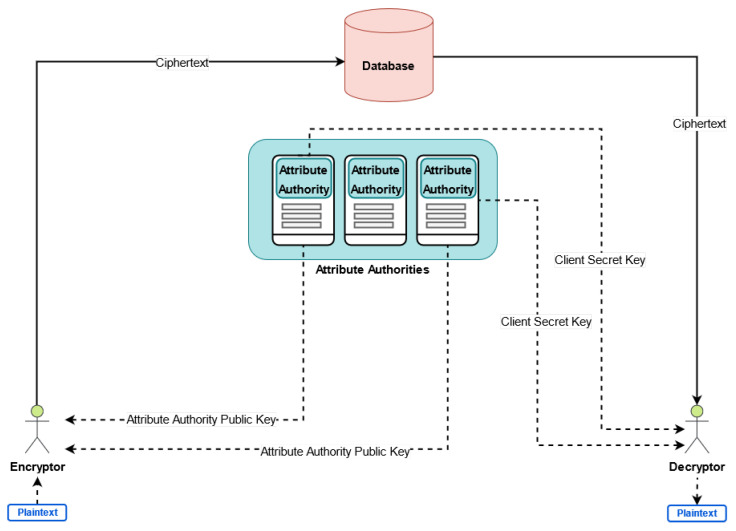
Framework of multi authority attribute based encryption.

Let us note that in large-scaled distributed environment, the traditional ABE schemes with single attribute authority might face some efficiency and scalability issues. In order to reduce the workload and attack risk of single authority, Chase [37] proposed Multi-Authority Attribute Based Encryption (MA-ABE), which has *n* attribute authorities and one central authority, which does not monitor any attributes. Furthermore, Lewko and Waters [38] proposed the Decentralized Attribute Based Encryption (DABE) that removes the centralized authority, without any requirement for global coordination between distributed attribute authorities. As it is illustrated in Figure 1, A user gets the public keys of the relevant attribute authorities and encrypt the plaintext using these public keys. On the other hand, a user wants to decrypt the ciphertext has to prove its attributes to the relevant attribute authorities and asks for corresponding decryption key. The decentralized ABE scheme [38] is composed of the following five algorithms: Global Setup(), Attribute Setup(), Key Generation(), Encrypt() and Decrypt().

Global Setup(): It is a random algorithm to initialize the public parameters, that are the bilinear group, the generator and the random hash function that maps the global user identifier to an element in the bilinear group.

Attribute Setup(): Each attribute authority chooses two random exponents α,β for each attribute that it handles, keep them as its secret key and publishes $e{\left(g,g\right)}^{\alpha }$ and $g^{\beta }$ as its public key.

Key Generation(): It is an algorithm conducted by an attribute authority and generates secret key for users. It takes the authority’s secret key and global user identifier as an input and generates the secret key for the user regarding its specific attribute.

Encrypt(): This algorithm is run by a sender. The sender will take the attributes for each authority, system public parameters and plain-text of a message, then generate the ciphertext as output.

Decrypt(): This algorithm is conducted by a user. It takes the ciphertext as the input, which encrypted under specific access structure. The user can decrypt the ciphertext only if it has the required set of attributes.

The security of the decentralized ABE system can be defined with the help of the following game simulated by a challenger and an adversary Adv:

Setup phase: The challenger runs the Global Setup and Attribute Authority Setup algorithms and sends the generated public parameters and the public key of the authorities to Adv.

Secret key queries phase: Adv repeats the secret key queries as many as possible corresponding to the sets of attributes: $A_{Adv_{1}}$,...,$A_{Adv_{n}}$. Then the challenger responses the corresponding secret keys to the adversary.

Challenge phase: The adversary Adv gives two equal length messages $M_{0},M_{1}$, respectively. Meanwhile, it gives a challenges discovery policy dPolicy such that none of sets of attributes from Secret key queries phase satisfy this structure. Then, the challenger flips a random coin b∈0,1, encrypts message $M_{b}$ under the chosen access structure dPolicy, and sends the ciphertext to Adv.

More secret key queries phase: The secret key queries phase is repeated with the restriction that the attribute sets cannot satisfy dPolicy.

Guess: The adversary Adv outputs a guess $b^{\prime }$. We assume Adv is successful if $b=b^{\prime }$, i.e., it finds out which message was encrypted in the Challenge phase correct resource out of the two resources $res_{0}$ and $res_{1}$, rather than being able to decrypt the address of the resource correctly. We define the advantage of Adv in this game as $Pr\left[b^{\prime }=b\right]-\frac{1}{2}.$

**Definition** **6.** *A distributed CP-ABE system is secure if every PPT adversary Adv has at most negligible advantage in the above game.*

The security of the scheme of Lewko and Waters based on the hardness of the General Subgroup Decision problem in Definition 4:

**Theorem** **1** (Lewko and Waters [38]). *If the General Subgroup Decision problem is hard, then the DABE system in [38] is secure.*

## 4. Model Description

One of the requirements of the models that are designed for IoT is the avoidance of single point of failures as it can be a centralized service, even if implemented using redundancy and replication. The proposed model keeps the resources discoverable only by clients that have certain attributes without relying on a centralized entity by following the general system trend of fog/edge computing. Attred is based on structured p2p schemes [39] and implements the RDHT [32]. In the following the proposed model is explained.

There are four main sets in Attred: set of clients (C), set of objects (O), set of gateways (W) and set of attribute authorities (AA). The finite set C consists of the IoT clients in the network, and each IoT client c∈C has a set of attributes. These attributes may be its location, its employment status and so on. An object o∈O is any device in the IoT network with proper computational power that handles a resource res. Each resource res is defined in the network by a number of attributes that indicates its different properties, e.g., its type, its provided service, and so on. Members of *C* and *O* are connected to different IoT gateways in W. We assume that there is a secure link between the members of *C* and *O* on one hand, and their directly connected IoT gateways on the other hand. A gateway *w* and depending on its location may handle different number of nodes from sets C and O. The finite set AA consists of the attribute authorities in the network. An attribute authority $AA_{i}\in \mathrm{AA}$ is an independent entity responsible to generate the public/private keys of attribute *i*. There is no centralized entity in AA, and any entity that manage an attribute can have an attribute authority of that specific attribute as part of AA. Attred utilizes the defined workflow of Kademlia [17] implementation of p2p scheme with RDHT [32] that provides a structured method of addressing and discovery of the peers. The members of W represent the peers in the RDHT overlay.

### 4.1. Model Features

The proposed model of resource discovery provides the following features:

- Scalability: Attred is based on the RDHT as an overlay for managing the edge and fog nodes in the system. Due to adopting the DHT technology, the added overhead increases logarithmically which makes it scalable.
- Attribute based discovery: The resources in Attred can be discovered based on their attributes. Therefore, there is no need to know the exact identifier of a resource to be able to discover it.
- Location aware discovery: Attred creates an overlay of IoT gateways divided logically into multiple region sets in RDHT and the resources can be registered and discovered based on their physical locations.
- Attribute based access control: The resources in the system are able to define the set of attributes that a client should have in order to being able to discover the registered resources. This feature allows some resources to being discoverable only by a predefined subset of clients based on their attributes.
- Discoverability: Attred is able to be fully integrated with the Distributed Address Table (DAT) [40] as parts of its system to allow discovering and accessing all resources in the network including those behind the Network Address Translator (NAT). This is a crucial requirement for IoT environment with huge number of nodes behind a firewall/NAT.
- Responsibility Definition: Attred clearly defines a specific node or a distinct subset of nodes that are responsible for registering any resource in the system, without relying on any centralized organizing entity. Therefore, during the discovery process the same distributed subset of nodes can be used to discover the required resources.

### 4.2. Security Model

Before defining the security properties we need to define assumptions regarding the participants of Attred.

**Definition** **7** (Semi-honest entity). *The semi-honest entity (honest-but-curious entity) in the system follows the protocol properly, but it might store the received data locally in an attempt to get more information from the stored data.*

**Definition** **8** (Malicious entity). *The malicious entity in the system does not follow the protocol properly. It might passively eavesdrop the messages or actively take an action to forge an identity of another node, modify a message or deny services to other nodes in the system.*

In the point of view of a registered resource and a client that issues a discovery request, their directly connected gateways are considered semi honest nodes. Other members in the RDHT overlay might be considered malicious nodes. The proposed model is assumed to achieve computational security, i.e., every Probabilistic Polynomial-Time (PPT) adversary can break the security properties with negligible probability only. The proposed model has to satisfy the following security properties:

- Discovery correctness: any semi-honest client that has the required attributes defined in the discovery policy can discover the address data of the resource.
- Discovery soundness: every PPT adversary and without the required attributes defined in the discovery policy can discover the resource with negligible probability only.
- Resource privacy: every PPT adversary can learn the relationship between the address data and a resource, without having required attributes defined in the discovery policy with negligible probability only.
- Client privacy: every PPT adversary can learn the private attributes in discovery requests issued by members of C with negligible probability only.

Note that in our proposed scheme the address data are encrypted with a DABE scheme, hence the ability to discover the address data means the ability to decrypt a ciphertext related to the address data. Since our proposed scheme based upon the protocol [38], we consider it in the above-mentioned indistinguishability sense (Definition 6).

Let H(.) be a collision resistant one-way hash function with *d* bits message digest and $Sign_{w}\left(m\right)$ be a digital signature algorithm for message *m* generated by w∈W gateway.

### 4.3. Overlay Description

Attred creates a RDHT overlay to organize the members of W and AA. The RDHT overlay is divided into different region sets, each of them consists of a region representative and a number of local regions. The first region set in Attred consists of two regions, attributes authorities region and general region. Assume that a collision-resistant one-way hash function H(.) is used in RDHT that outputs a *d* bits digest. The members of AA are positioned in the attributes authorities region by hashing the attribute that each attribute authority is responsible for. The identifier of the members of AA starts with *d* zeros, followed by the output of the hash function. All IoT gateways in the system reside in the general region, regardless of their physical locations. The members of W are positioned in the general region by hashing the unique information of the nodes (e.g., IP addresses), and append that to a sequence of d−1 zeros and a single bit 1.

**Figure 2:**
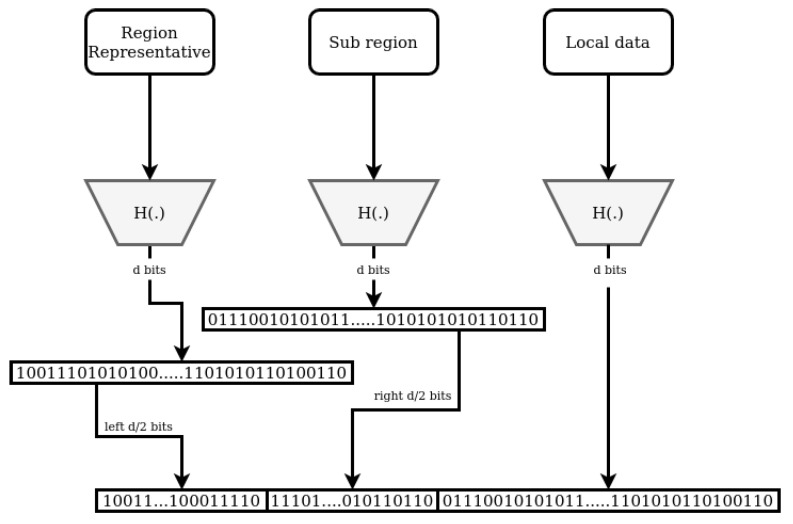
Identifier generation in RDHT.

The rest of the regions in RDHT are divided into different regions sets based on different locations. In addition to the general region, the members of W are positioned in different local regions based on their physical locations. The approach to create the identifier of an IoT gateway residing in a local region (in addition to the general region) is illustrated in Figure 2. Each RDHT identifier consists of two concatenated parts, region identifier and local identifier. Each region set has a region representative that represents that region and number of local regions (i.e., sub regions). To create the region part of an identifier of a peer w∈W, the information of the region representative of that region set is fed to the hash function and the first left d/2 bits of the output represents the first d/2 bits of the generated region part of the identifier. The given input information of the locations can be represented by human readable names of regions or a specific prefix of latitude/longitude data. The local region information is then fed to the hash function and the last d/2 bits are taken that will represent the last d/2 bits of the generated region identifier. The nodes in the representative region itself, will have the last d/2 bits all set to zero. Because of the Avalanche effect property [41] of the hash function algorithms, each subset of a generated digest by the hash function should be affected equally as any other subset of the digest. Therefore, generating the region identifier by taking d/2 bits from the *d* bits digest of representative region and d/2 bits from the *d* bits digest of the local region should not affect the randomness of the generated identifier.

The remaining part of the identifier of a node, i.e., the local identifier, is generated by hashing the information of the IoT gateway (e.g., its IP address). As a result, the identifiers of the nodes in the same local region share the same *d* prefix bits. Additionally, the identifiers of the nodes in all local regions of a region set share the same d/2 prefix bits. Each node in RDHT overlay has *d* lists of the *k*-buckets [17] that include the addresses to the nodes in the same region. In addition, each node in any region of a region set should keep d/2 lists of the k-buckets that include access addresses to all representative regions in all region sets in RDHT, including the attribute authorities and general regions. The nodes in the representative region of any subset, keep d/2 lists of the k-buckets that include access addresses to all regions in the region set. As a result, the nodes in the representative regions have 2d lists and all other nodes in any region in RDHT have *d* lists. This mechanism ensures the ability to retrieve the address data of any node in the RDHT overlay.

### 4.4. System Setup

**Figure 3:**
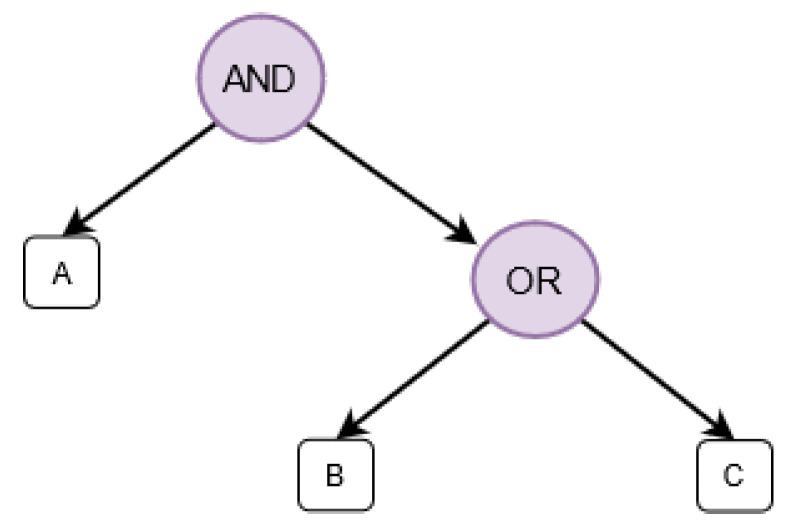
An example of a discovery policy.

Assume that each client cln∈C has a unique global identity, namely $UI_{cln}$. Let $A_{cln}$ denotes the set of the attributes of a client cln, $A_{res}$ donates the set of attributes that describes a resource res, and dPolicy denotes a Boolean formula of discovery policy that defines the required attributes to be able to discover a resource res in Attred. An example of dPolicy is shown in Figure 3 where the clients should have attribute “A” in addition to either attribute “B” or “C” to be able to discover this specific resource.

Our model uses a decentralized ABE scheme [38], specifically, in the secret key generation, encryption and decryption steps in Section 4.6, Section 4.7 and Section 4.8. During system setup a set of global parameters (GP) is generated. To generate GP, first a bilinear group G of composite order is selected, and then a generator *g* of a subgroup of G is chosen. In addition, the hash function $H_{G}\left(.\right):{\left\{0,1\right\}}^{*}\to G$ is defined that maps the unique global identities of the clients to a member of G.

In system setup the RDHT overlay will be created. The IoT gateways are the peers in the overlay. Each gateway w∈W and upon joining the network randomly generates an identifier to be able to be part of RDHT. An identifier in RDHT is generated using a collision-resistant one-way hash function $H:{\left\{0,1\right\}}^{*}\to {\left\{0,1\right\}}^{d}$ as discussed in Section 4.3. A subset of objects and clients are connected to each gateway w∈W.

### 4.5. Attribute Authority Registration

After choosing the global parameters in GP, the attribute authorities can be setup. At any given time, each organization that is responsible for an attribute *i* can setup an attribute authority $AA_{i}\in \mathrm{AA}$ and be part of the set of attributes authorities. In case that the attribute authority wants to follow a privacy preserving approach of its clients, it can use the collision-resistant one-way hash function to generate the hash values of the attributes that is responsible for. These hash values can be used without revealing the pre-image that includes the values of the attributes. In Attred, there is no centralized authority and there is need for any cooperation between attributes authorities. Therefore, any organization can setup its own attribute authority independently. To do so, an organization that handles an attribute *i* (or H(i) if the attribute authority follows the privacy preserving approach), chooses two random exponents $\alpha_{i},\beta_{i}\in Z_{p}$ that defines its pair of private key. Then, it publishes $\left\{PA_{i}=e{\left(g,g\right)}^{\alpha_{i}},PB_{i}=g^{\beta_{i}}\right\}$ as its public key. The set $P_{AB}$ includes all the public pairs PA,PB of the attribute authorities in the system.

### 4.6. Client Secret Key Generation

We assume that each client cln∈C in the system has a unique global identity ($UI_{cln}$). Each client has a set of attributes $A_{cln}$. These attributes can be its occupation, its organization and so on. If a client wants to keep some of its attribute private, it can use the collision-resistant one-way hash function to generate the hash values of its attributes. These hash values can be used without revealing the pre-image that includes the attributes. This has to be done in agreement with the attribute authority. We assume that for private attributes the rainbow attack [42] is hard. To create a secret key of client cln for an attribute $i\in A_{cln}$, it has to contact the relevant attribute authority and after proving its identity, a secret key $sk_{i,cln}$ (or $sk_{H(i),cln}$ in case that the attribute authority follows a privacy preserving approach) will be generated by the attribute authority $AA_{i}\in \mathrm{AA}$ as in (1).

(1) $$sk_{i,cln}=g^{\alpha_{i}}{\left(H_{G}\left(UI_{cln}\right)\right)}^{\beta_{i}}$$

The secret key $sk_{i,cln}$ is tied to the client cln using its unique global identity $UI_{cln}$. This feature prevents number of adversaries from combining their attributes to be able to discover a resource that cannot be discovered by one of them.

### 4.7. Resource Registration

**Figure 4:**
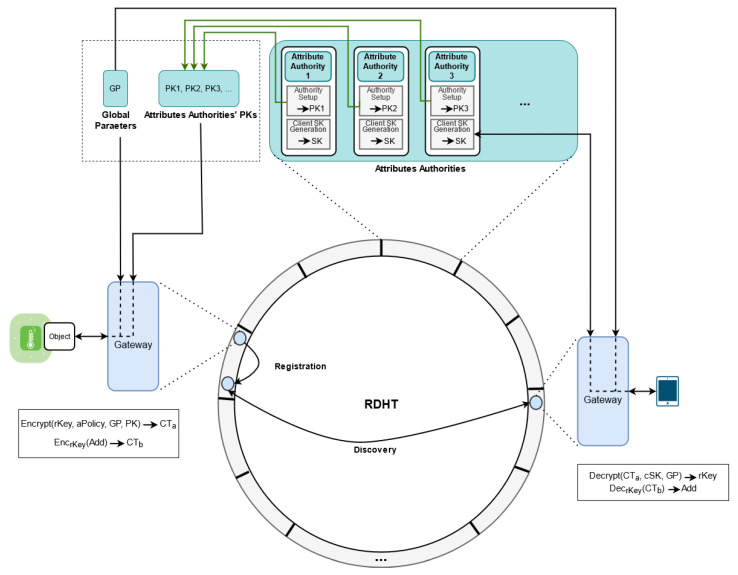
Resource registration and discovery in Attred.

A resource res has its unique address and a set of attributes ($A_{res}$) which describes it in the network. These attributes can be its location, its type and so on. The main task of a resource discovery is to allow discovering the address data add of the resource res based on its attributes. Additionally, the address data add have to be retrieved only by clients whose attributes satisfy the defined discovery policy (dPolicy) during registration. Figure 4 illustrates the process of resource registration and discovery in Attred.

To register a resource res by an object *o* in the network, the object has to define its attributes (i.e., $A_{res}$), the discovery policy that defines the required attributes of the clients that are able to access the address of res (i.e., dPolicy), the ownership data, the registration level and either locally perform the required encryption and resource registration or send them to the directly connected gateway to perform the required encryption and resource registration. We assume that the address data of the resource res can be represented as a group member in G. The add value will be encrypted based on the discovery policy dPolicy and stored in Attred as a tuple of (tag,ownership,dPolicy,cAddress). If add cannot be represented as a group element in G, then a random member rKey can be chosen and encrypted based on ABE. Then, the address data of the resource res are encrypted based on a symmetric encryption using the random key rKey.

The tag in the tuple (tag,ownership,dPolicy,cAddress) is generated as a result of hashing each of the attributes that describes the resource res using a collision-resistant one-way hash function H(.). During resource registration, the object *o* generates a random number $r_{ownership}$ and adds its hashed value as a later proof of ownership of the generated tuple. Revealing the pre-image of the hash value (i.e., $r_{ownership}$) guarantees the ability of proving the ownership of the tuple that will used during updating or removing it from the system. The dPolicy parameter describes the Boolean formula of the required attributes and rules to discover and decrypt the registered resource address add. The cAddress parameter consists of the address data of the resource res that is encrypted based on ABE.

There are two registration levels that can be defined in the process of registration, namely local and general. A resource in Attred can be registered locally in the same region. Additionally, the resource can also be registered in the general region of RDHT. The advantage of registering a resource locally is its low required time for registration compared to registering a resource in the general region. This is due to the fact that the peers in the same local region are physically close which makes the communication relatively faster. In addition, discovering the registered resources by the clients in the same regions takes less time as well. The disadvantage of registering a resource locally is that it requires selecting the specific region during the discovery process by the clients. Without this knowledge, the clients are not able to discover the locally registered resources. In contrast, the registered resources in the general region can be discovered regardless of their physical locations.

**Figure 5:**
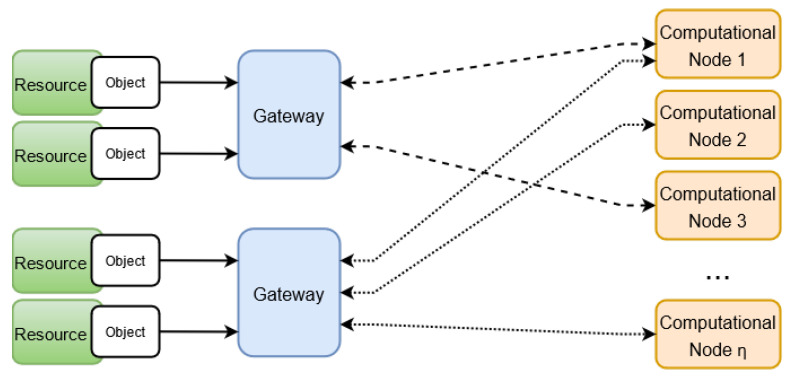
Computational nodes.

The direct peer in the RDHT overlay (i.e., The IoT gateway that the object *o* is connected to) stores these tuples locally for a specific time depending on the caching expiry parameters. Additionally, the close peers in the targeted regions in RDHT to tag parameter of the generated tuples are responsible for storing (tag,ownership,dPolicy,cAddress) tuple. Attred does not depend on any specific distance function to compute the closeness (dst), and it can be any particular distance function. Metrics such as bitwise exclusive or (xor) [17] can be used to compute dst value. A subset of W will indicate the gateways in a specific region in RDHT that have smallest dst value with the tag parameter of the tuple related to the resource res. The cardinality of this subset depends on the replication factor. This factor indicates the number of peers in *w* that are responsible for storing a replica of the tuple (tag,ownership,dPolicy,cAddress). The process of registering a resource res in the network consists of seven steps:

- Tag Definition and Generation: the object *o* that wants to register its resource res in the network defines set of attributes that describes the resource res (e.g., its location, its type, etc.). Based on these attributes and using the hash function H(.), it generates the tags that represent the first part of the final (tag,ownership,dPolicy,cAddress) tuples.
- Ownership Generation: the object *o* generates a random number $r_{ownership}$, and adds its hashed value using the hash function H(.) as a later proof of ownership of the generated tuple.
- Discovery Policy Definition: in this step the object *o* defines the Boolean formula dPolicy in the final (tag,ownership,dPolicy,cAddress) tuples, the represents the set of attributes of clients that are eligible to discover the resource res. This step is conduced locally, as we need to let the resources themselves define the legitimate clients.
- Registration Level Definition: The object *o* defines the regions in which the resource has to be registered in. It can be local level, general level or both. The local level results in registering the resource in the same region, while the general level results in registering the resource in the general region, regardless of its physical location.
- Resource Address Encryption: The object *o* after defining and generating the tags and the attributes can encrypt the resource address. If the object *o* does not have the required computational power, the address data add can be encrypted in the directly connected gateway *w*. In both cases (encrypting directly in the object or in the gateway), Attred proposes an approach of securely distributing parts of the heavy computation steps to other nodes. Lets assume that the directly connected gateway *w* receives the set of tags, the address data, and the attribute set from the object *o*. Taking the address data (add) of res, the system global parameters (GP) and set of public keys of the attribute authorities ({PK}), the gateway *w* generates the cAddress parameter of the tuples. The cAddress parameter includes a main component cAddress(0), the encrypted address data using ABE and a set of three components ($cAddress\left(i_{1}\right),cAddress\left(i_{2}\right),cAddress\left(i_{3}\right)$) for each attribute *i* in the discovery policy. The later set of three components are used by the clients to be able to decrypt the address of a discovered resource. The gateway *w* first converts the Boolean formula of the discovery policy dPolicy to a linear secret sharing scheme (LSSS) matrix M(dPolicy). As instance, the dPolicy in Figure 3 will be converted to $M\left(dPolicy\right)=\left(\begin{array}{cc} 1 & 1\\ 0 & -1\\ 0 & -1 \end{array}\right)$. The gateway *w* then chooses a random secret $s\in Z_{p}$, and generates a vector γ (i.e., an ordered finite list of numbers) where its length is equal to number of columns in M(dPolicy), its first element is set to *s*, and the rest elements chosen randomly from $Z_{p}$. This vector ensures that only clients with required attributes can get the random secret *s* that is required to decrypt the address data. It also generates a vector ω where its length is equal to number of columns in M(dPolicy), its first element set to zero, and the rest elements chosen randomly from $Z_{p}$. This vector ensures that no two clients can combine their attributes in an attempt to decrypt the address data.
  Additionally, it chooses three parameters $r_{i},\gamma_{i},\omega_{i}$ for each attribute *i*, i.e., each leaf in the discovery policy. $r_{i}$ is selected randomly from $Z_{p}$. The gateway *w* computes $\gamma_{i}$ for each attribute *i* in the discovery policy using (2), where $M{\left(dPolicy\right)}_{i}$ denotes the *i*th row in M(dPolicy).
  (2) $$\gamma_{i}=M{\left(dPolicy\right)}_{i}.\gamma$$
  The gateway *w* computes $\omega_{i}$ for each attribute *i* in dPolicy using (3).
  (3) $$\omega_{i}=M{\left(dPolicy\right)}_{i}.\omega$$
  The address data add of the registered resource is encrypted as (4) and the three parameters of an attribute *i* in the discovery policy dPolicy is computed as in (5)–(7).
  (4) $$cAddress\left(0\right)=\left(add\right)e{\left(g,g\right)}^{s}$$
  (5) $$cAddress\left(i_{1}\right)=e{\left(g,g\right)}^{\gamma_{i}}{PA_{i}}^{r_{i}}$$
  (6) $$cAddress\left(i_{2}\right)=g^{r_{i}}$$
  (7) $$cAddress\left(i_{3}\right)={PB_{i}}^{r_{i}}g^{\omega_{i}}$$
  There are a number of public and independent computational nodes in the system as illustrated in Figure 5. These nodes are assumed to be independent and semi-honest computational nodes that are able to perform heavy computational operations. The resource registration and discovery can be done without involving the computational node, but using those nodes improves the resource registration time. Assume that the nodes performing the encryption wants to improve the registration time and has connections with η independent semi-honest computational nodes, as instance cloud servers, such that η⩾2. The steps in Equations (4)–(7) can be distributed based on the additive secret sharing and computed using (12)–(15) instead, as following: The node *w* first chooses η random additive shares $s_{1},\ldots ,s_{\eta }\in Z_{p}$ such that their summation is equal to the random parameter $s\in Z_{p}$.
  (8) $$s\equiv \sum_{j=1}^{\eta }s_{j}$$
  Then, for each attribute *i* in the discovery policy dPolicy, the node *w* also chooses random additive shares for $\gamma_{i},r_{i}$ and $\omega_{i}$ satisfying (9)–(11), respectively. These are done to be able to distribute the computation overhead of heavy exponentiation among the η semi-honest nodes, without revealing the secret values.
  (9) $$\gamma_{i}\equiv \sum_{j=1}^{\eta }\gamma_{ij}$$
  (10) $$r_{i}\equiv \sum_{j=1}^{\eta }r_{ij}$$
  (11) $$\omega_{i}\equiv \sum_{j=1}^{\eta }\omega_{ij}$$
  After selecting the random numbers, each of the η computational nodes receives $s_{j},$ and a set $\left(attribute_{i},\gamma_{ij},r_{ij},\omega_{ij}\right)$ for each attribute *i*. A node *j* and after receiving the set, computes $e{\left(g,g\right)}^{s_{j}}$ and $cAddress{\left(i_{1}\right)}_{j},cAddress{\left(i_{2}\right)}_{j},cAddress{\left(i_{3}\right)}_{j}$ using (5)–(7), respectively. Then, the node *j* sends the results back to *w*. The node *w* and after receiving the results from all η nodes, starts computing the final cAddress parameter using (12) for cAddress(0) and (13)–(15) for the three components ($cAddress\left(i_{1}\right),cAddress\left(i_{2}\right),cAddress\left(i_{3}\right)$) of each attribute *i* in the discovery policy.
  (12) $$cAddress\left(0\right)=\left(add\right)\prod_{j=1}^{\eta }cAddress{\left(0\right)}_{j}=\left(add\right)\prod_{j=1}^{\eta }e{\left(g,g\right)}^{s_{j}}$$
  (13) $$cAddress\left(i_{1}\right)=\prod_{j=1}^{\eta }cAddress{\left(i_{1}\right)}_{j}=\prod_{j=1}^{\eta }e{\left(g,g\right)}^{\gamma_{ij}}{PA_{i}}^{r_{ij}}$$
  (14) $$cAddress\left(i_{2}\right)=\prod_{j=1}^{\eta }cAddress{\left(i_{2}\right)}_{j}=\prod_{j=1}^{\eta }g^{r_{ij}}$$
  (15) $$cAddress\left(i_{3}\right)=\prod_{j=1}^{\eta }cAddress{\left(i_{3}\right)}_{j}=\prod_{j=1}^{\eta }{PB_{i}}^{r_{ij}}g_{j}^{\omega_{i}}$$
  It is noteworthy to mention that if the defined discovery policy dPolicy by the object will not change, the distributed computation can be done in advance and stored locally regardless of the address data of the resource. This means that in this case the computational nodes do not have to be online at the time of resource registration.
- Tuple Signing: The tuples are constructed as (tag,ownership,dPolicy,cAddress) in which tag is the hashed value of an attribute in $A_{res}$ of the resource, ownership is used later to proof the ownership of this tuple, dPolicy is the the discovery policy that defines the required attribute set of the clients that can discover this resource, and cAddress is the encrypted address data add of the resource. These tuples of the resource res are signed $Sign_{w}\left(tuple\right)$ by the gateway *w*.
- Resource Registration: Finally, the gateway *w* puts each of the generated and signed tuples in the corresponding peer in RDHT that is responsible to store this tuple (i.e., its identifier is the closet to the tag parameter in the tuple). The registration is done in the same local region, in the general region or in both regions, based on the request of the object *o*.

### 4.8. Resource Discovery

Any client cln∈C can search for resources based on their attributes. The retrieved data (i.e., the address of the resource) can be read only if the client cln has the relevant attributes required to decrypt the retrieved resource address, i.e., if the Boolean discovery policy dPolicy taking $A_{cln}$ returns true. A client cln∈C starts the discovery process by sending a lookup request including its attributes (to find the resource that can be discovered), the required set of resource attributes (to find the exact resources) and the targeted region to the gateway *w* that is directly connected to. The gateway *w* after receiving a discovery request from a client cln generates the appropriate tags for the lookup process in the overlay based on the received request from the client. The retrieved address of the resource from the resource discovery procedure can be accessed only by having the appropriate attributes by the client cln. The discovery process includes the following main steps:

- Query Generation: First, the client cln sends its attribute, the required attributes of the resource to be discovered in the network and the targeted region to the directly connected gateway *w*. The gateway *w* generates set of tags, by hashing the required attributes of the discovered resources in the received request from cln.
- Lookup: in this step, the gateway *w* issues the lookup process in the targeted region in RDHT overlay to retrieve the specific tuples based on the required tags and the set of attributes of the client $A_{cln}$. The later information, i.e., the attributes of the client, is optional and can be ignored to hide the attributes of the clients. The discovery can be local, intra-regional or regional. The local discovery is done to discover a resource that is registered in the same region that the client belongs to. In this case, both source and destination gateways share the same *d* prefix bits, where *d* is the length of the output of the used hash function H(.). The intra-regional discovery is done to discover a resource that is registered in a different region of the same region set. This means the the identifiers of both source and destination of the lookup share same d/2 prefix bits. The regional discovery is done to discover a resource that is registered in a region that belongs to a different region set, or a general resource regardless of its physical location. The recipient nodes in RDHT first filters the resources based on the required tags, and then checks the dPolicy parameter of the filtered tuples and get the final set of tuples based on the required attributes in the dPolicy parameter of each tuple and the attributes of the client cln. If $A_{cln}$ has not been sent in the request, all results returned back to the gateway *w*. The result $R_{i}$ of each of the lookup operations is a set of data parameters that indicates the resources with the specific attribute *i*.
- Result Verification: after receiving the digitally signed results $R_{0},R_{1},\cdots ,R_{n}$ of the required attributes, they will be verified and the intersected members of sets $R_{discovery}=R_{0}\cap R_{1}\cap \cdot \cdot \cap R_{n}$ will be gathered. In addition, the dPolicy parameter of the generated set $R_{discovery}$ will be checked locally and filtered based on the required attributes in the dPolicy parameter of each tuple in $R_{discovery}$ and the attributes of the client cln (i.e., $A_{cln}$). Some ranking approaches to $R_{discovery}$ might be applied in this step as well.
- Resource Address Discovery: finally, depending on the attributes of client cln and dPolicy parameter in the retrieved tuples, the resources in $R_{discovery}$ will be returned to cln and the retrieved cAddress of the resource in the tuples will be decrypted based on the attributes $A_{cln}$ of client cln. For each attribute *i* in dPolicy parameter of the tuple, and using the $cAddress\left(i_{1}\right),cAddress\left(i_{2}\right)$ and $cAddress(i_{3})$ components in the cAddress and its relevant secret key $sk_{i,cln}$ of that attribute, the client computes the value in (16). The results of the retrieved values of all attributes is used in (17) to retrieve the pairing value of the random secret *s*.
  (16) $$\begin{array}{c} es_{i}:=cAddress\left(i_{1}\right).e\left(H_{G}\left(UI_{cln}\right),cAddress\left(i_{3}\right)\right)\\ /e\left(sk_{i,UI_{cln}},cAddress\left(i_{2}\right)\right)=e{\left(g,g\right)}^{\gamma_{i}}e{\left(H_{G}\left(UI_{cln}\right),g\right)}^{\omega_{i}} \end{array}$$
  (17) $$e{\left(g,g\right)}^{s}=\prod_{i=1}^{\left|dPolicy\right|}{es}_{i}^{c_{i}}$$
  where $c_{i}$ is a constant in $Z_{p}$ such that $\sum_{i=1}^{\left|dPolicy\right|}c_{i}M{\left(dPolicy\right)}_{i}$ returns a vector with the first element only set to one, and the rest set to zero. Finally, the address data of the resource is recovered by decrypting the address data as in (18).
  (18) $$add=cAddress\left(0\right)/e{\left(g,g\right)}^{s}$$

### 4.9. Resource Update and Removal

The tuples of the registered resources remain in Attred based on the caching expiry parameter. In addition to that, an object is able to update the data or remove its registered resource from Attred by issuing a request including the pre-image of the ownership field in the added tuple. The request is signed by the directly connected node in Attred, *w* and is sent to the corresponding node in Attred. After checking the ownership of the tuple (i.e., $H(r_{ownership})=ownership$), the requested tuple is updated by a new tuple or removed from Attred based on the received request.

## 5. Evaluations

In this section, we study and analyze Attred. First we prove Attred is secure and satisfies the discussed security properties under some assumptions. Then, we discuss access control, privacy and availability in Attred. We also study its scalability and efficiency in terms of complexity analysis. Finally, we evaluate the resource registration in RDHT overlay, the improvements in the distributed resource registration process and the efficiency of Attred compared with some resource discovery models.

### 5.1. Security Analysis

In this section, we prove the security properties (discovery correctness, discovery soundness, resource privacy and client privacy) of Attred.

**Theorem** **2.** *If an object o and a client cln follow the Attred protocol for registration and discovery, and cln receives uncorrupted cAddress parameter, by having required attributes the system satisfies discovery correctness.*

**Proof.**  Given a bilinear group G of prime order *p*, a generator *g* of G, $\alpha_{i},\beta_{i}\in Z_{p}$ the private key of attribute authority *i* kept private by the authority and $e{\left(g,g\right)}^{\alpha_{i}}$, $g^{\beta_{i}}$ its published public key, assume an object *o* registers a resource by generating the following: a discovery policy dPolicy and its LSSS matrix M(dPolicy), a random secret $s\in Z_{p}$, a vector γ=(s,⋯) and a vector ω=(0,⋯). It also computes $r_{i}\overset{R}{\leftarrow }Z_{p}$, $\gamma_{i},\omega_{i}$ for each leaf *i* in dPolicy. The published address consists of $cAddress\left(0\right)=\left(add\right)e{\left(g,g\right)}^{s}$, and $cAddress\left(i_{1}\right)=e{\left(g,g\right)}^{\gamma_{i}},cAddress\left(i_{2}\right)=g^{r_{i}}$ and $cAddress\left(i_{3}\right)={PB_{i}}^{r_{i}}g^{\omega_{i}}$ for each leaf *i* in dPolicy.

In order to get add, the client should retrieve add which can be done by knowing the value of the random secret *s*. Assume that each client has a unique global identity $UI_{cln}$ and a client cln has the secret keys $sk_{i,cln}=g^{\alpha_{i}}{\left(H_{G}\left(UI_{cln}\right)\right)}^{\beta_{i}}$ of some *i* in dPolicy that represents a valid subset of dPolicy, it computes the following:

$$es_{i}=\frac{Address\left(i_{1}\right).e\left(H_{G}\left(UI_{cln}\right),cAddress\left(i_{3}\right)\right)}{e(sk_{i,UI_{cln}},cAddress\left(i_{2}\right))}=$$

$$=\frac{e{\left(g,g\right)}^{\gamma_{i}}e{\left(g,g\right)}^{\alpha_{i}r_{i}}.e\left(H_{G}\left(UI_{cln}\right),g^{\beta_{i}r_{i}}g^{\omega_{i}}\right)}{e(g^{\alpha_{i}}{\left(H_{G}\left(UI_{cln}\right)\right)}^{\beta_{i}},g^{r_{i}})}=e{\left(g,g\right)}^{\gamma_{i}}e{\left(H_{G}\left(UI_{cln}\right),g\right)}^{\omega_{i}}$$

The client then chooses $c_{i}\in Z_{p}$ for each row in M(dPolicy) such that (1,0,⋯,0)=$=\sum_{i=1}^{\left|dPolicy\right|}c_{i}M{\left(dPolicy\right)}_{i}$. Since cln has the required attributes, it can choose such constants. raising the last computed result $es_{i}$ to the power of these constants and adding all results together allow revealing the secret by removing all random variables in the first term of $es_{i}$ ($e{\left(g,g\right)}^{\gamma_{i}}$) and leaving only the first element (i.e., γ(0)=s), and removing all random variables in the the second term of $es_{i}$ ($e{\left(H_{G}\left(UI_{cln}\right),g\right)}^{\omega_{i}}$) by revealing the secret value zero (since ω(0)=0). The add is computed as follows:

$$add=cAddress\left(0\right)/\prod_{i=1}^{\left|dPolicy\right|}({e{\left(g,g\right)}^{\gamma_{i}}e{\left(H_{G}\left(UI_{cln}\right),g\right)}^{\omega_{i}})}^{c_{i}}=cAddress\left(0\right)/e{\left(g,g\right)}^{s}.$$

□

**Theorem** **3.** *If an object o and a client cln follow the Attred protocol for registration and discovery, and the η computational nodes are independent semi-honest nodes, then the returned results from distributing the computations among those nodes satisfies discovery correctness as if calculating it locally.*

**Proof.**  Given a bilinear group G of prime order *p*, a generator *g* of G, $\alpha_{i},\beta_{i}\in Z_{p}$ the private key of attribute authority *i* kept private by the authority and $e{\left(g,g\right)}^{\alpha_{i}}$, $g^{\beta_{i}}$ its published public key, assume an object *o* registers a resource by generating the following: a discovery policy dPolicy and its LSSS matrix M(dPolicy), a random secret $s\in Z_{p}$, a vector γ=(s,⋯) and a vector ω=(0,⋯). It also computes $r_{i}\overset{R}{\leftarrow }Z_{p}$, $\gamma_{i},\omega_{i}$ for each leaf *i* in dPolicy. Assume there are η independent and semi-honest computational nodes. The object *o* chooses η random additive shares for $\gamma_{i},r_{i}$ and $\omega_{i}$ (Definition 1), for each leaf *i* in dPolicy.

Each of the η nodes receives $s_{j}$, and a set $\left(attribute_{i},\gamma_{ij},r_{ij},\omega_{ij}\right)$ for each attribute *i*. A node *j*, computes $cAddress{\left(0\right)}_{j}=e{\left(g,g\right)}^{s_{j}}$ and for each $attribute_{i}$ the values $cAddress{\left(i_{1}\right)}_{j}=e{\left(g,g\right)}^{\gamma_{ij}},cAddress{\left(i_{2}\right)}_{j}=g^{r_{ij}},cAddress{\left(i_{3}\right)}_{j}={PB_{i}}^{r_{ij}}g^{\omega_{ij}}$.

The object *o* computes cAddress(0) part of the final cAddress parameter the as follows: $cAddress\left(0\right)=\left(add\right)e{\left(g,g\right)}^{s}=\left(add\right)\prod_{j=1}^{\eta }cAddress{\left(0\right)}_{j}=\left(add\right)\prod_{j=1}^{\eta }e{\left(g,g\right)}^{s_{j}}$ and for each attribute *i* the set $cAddress\left(i_{1}\right)=e{\left(g,g\right)}^{\gamma_{i}}=\prod_{j=1}^{\eta }e{\left(g,g\right)}^{\gamma_{ij}}=\prod_{j=1}^{\eta }e{\left(g,g\right)}^{\gamma_{i}j}{PA_{i}}^{r_{ij}}$, $cAddress\left(i_{2}\right)=g^{r_{i}}=\prod_{j=1}^{\eta }g^{r_{i}j}$, and $cAddress\left(i_{3}\right)={PB_{i}}^{r_{i}}g^{\omega_{i}}=\prod_{j=1}^{\eta }cAddress{\left(i_{3}\right)}_{j}=\prod_{j=1}^{\eta }{PB_{i}}^{r_{ij}}g^{\omega_{ij}}$ are computed.

Since $s\equiv \sum_{j=1}^{\eta }s_{j}$, $\gamma_{i}\equiv \sum_{j=1}^{\eta }\gamma_{ij}$, $r_{i}\equiv \sum_{j=1}^{\eta }r_{ij}$, and $\omega_{i}\equiv \sum_{j=1}^{\eta }\omega_{ij}$ using the distributed computation returns the same result as if calculating it locally, without revealing the value of add. As of this stage, Theorem 2 is used to continue the proof of correctness. In addition, given the shares $s_{j},r_{ij},\gamma_{ij}$, and $\omega_{ij}$, each of the η computational nodes cannot get the final values of $s,r_{i},\gamma_{i},\omega_{i}$. □

**Theorem** **4.** *If the General Subgroup Problem is hard, then our model satisfies discovery soundness and resource privacy.*

**Proof.**  Since resource privacy is a consequence of discovery soundness, we prove the latter only. Let add be an address data and let Adv be a TTP adversary without the attributes defined in the discovery policy dPolicy. Then based on the reasoning mentioned in Section 4.2 Adv is able to discover add iff it can break the security of the DABE system in [38], an event which has negligible probability as a consequence of Theorem 1. This completes the proof. □

**Theorem** **5.** *If H(.) is a collision-resistant one-way hash function then the system satisfies client privacy.*

**Proof.**  Suppose that the discovery request containing the attribute parameter ATT of the client and the client has registered with the relevant attribute authority in a privacy preserving approach, ATT=H(att) where att is the attribute of the client. Let assume that the att is hard to be retrieved from ATT by the rainbow attack. Assume that the adversary Adv knows the ATT value. Since H(.) is a collision-resistant one-way function, Adv can get the value of att from ATT with negligible probability only. □

### 5.2. Discussion

Attred provides a resource registration and discovery in IoT. It solves the discussed issues of access control, privacy and availability. In the access control aspect, ABE shows its advantage in fine-grained access control. We proposed our solution based on ABE, supporting “AND” and “OR” gates when IoT object define the discovery policies. since only clients (with no cooperation with resources) need to verify their attributes in order to discover the address data of required resources, it fits perfectly the distributed IoT environment with huge number of resources.

In the privacy aspect, Attred addresses the problem of private information leakage. The address data of the resources are encrypted and the resources can only be discovered by the clients that have the required set of attributes. Since the discovery policy that includes this set of attributes is defined specifically by the object that handles the resource, it can assure its privacy by defining the attributes of the clients that can discover this registered resource. On the other hand, the attributes of the clients can be hold by their directly connected gateways and not included in the discovery request. Additionally, assuming that for private attributes the rainbow attack is hard and since each organization in Attred can establish its own attribute authority without any centralized authority or need for cooperation with other attribute authorities, the clients can hide their private attributes with an agreement with the attribute authorities. In this case, instead of the attributes their hash values will be used during resource discovery.

In order to reduce the workload and risk of single authority, we proposed a decentralized model to guarantee the availability aspect. The resources can be registered independently and directly without any need to direct communication with the attributes authorities or the clients. The addresses of IoT resources are stored in RDHT and some random peers in the RDHT are responsible to storing the registered addresses of the resources. A replication factor in RDHT assures that even if some peers failed, the addresses of the registered resources remain available in the system. The clients storing their secret keys locally can get the addresses of the required resources without any requirement of communication with the attributes authorities or the resources.

### 5.3. Complexity Analysis

Suppose that RDHT is divided into $N_{RegionSet}$ region sets. Let’s suppose that the three subsets $W_{R1},W_{R2},W_{R3}\subset W$ of IoT gateways represent the regions R1, R2 and R3 of RDHT overlay, with cardinality of $|W_{R1}|$, $|W_{R2}|$ and $|W_{R3}|$, respectively. Suppose that both R1 and R2 are in the same region set that includes $N_{RS1}$ local regions, and R3 is in a different region set. We discuss the complexity of the proposed model in four cases:

- Local Registering or discovering a resource in Attred ($C_{local}$).
- Registering or discovering a resource in the general region in Attred ($C_{general}$).
- Intra-regional discovering a resource in the same region set ($C_{intra}$).
- Regional discovering of a resource in a different region set than the client region ($C_{regional}$).

In all cases we assume there are *t* attributes for each resource or client in the system. Since getting the public and secret keys can be done once and at any given time, they are not part of the analysis. The client is supposed to have the required decryption keys, based on its approved attributes.

Registering a resource or discovering a resource by a client, locally in a region R1 in Attred is done by lookup the corresponding peer $w\in W_{R1}$ that is responsible for storing the tuple. It takes O(1) to create a tuple for each of the *t* attributes, and then $O(log(|W_{R1}\left|)\right)$ to find the corresponding peer to store the generated tuple. Therefore, registering a resource in Attred depends on the number required attributes, and number of nodes in generated overlay and is equal to $C_{local}=O(log(|W_{R1}{|}^{t}))$.

Since all nodes in Attred are part of the general region of RDHT, registering or discovering a resource in the general region (i.e., regardless of its physical location) takes $C_{general}={O(log(|W|}^{t}))$. If the client and the discovered resource are in regions R1 and R2 that are in the same region set including overall $N_{RS1}$ local regions, assuming having *t* attributes, the discovery access time takes $C_{intra}=O(log(N_{RS1}|W_{R2}{|}^{t})$ and is done in two stages. Firstly, it takes $O(log\left(N_{RS1}\right))$ to reach the target region (i.e., R2) and then it takes $O(log(|W_{R2}\left|)\right)$ for each of the *t* attributes to discover the required resource by reaching the specific responsible node in target region R2.

Discovering a resource in region R2 by a client belongs to region R3 that is in a different region set rakes $C_{regional}=O(log(N_{RegionSet}N_{RS1}|W_{R2}{|}^{t}))$ and is done in three stages. Firstly, accessing the representative region of the region set that the target region R2 belongs to takes $O(log\left(N_{RegionSet}\right))$ based on the number of available region sets. Then, reaching the region R2 takes $O(log\left(N_{RS1}\right))$. Finally, for each of the *t* attributes, it takes $O(log(|W_{R2}\left|)\right)$ to perform a lookup and discover the required resource by reaching the specific responsible node in target region R2.

### 5.4. Performance Analysis

The network latency has been taken into consideration for measuring the performance of RDHT overlay in Attred. Table 2 shows the assumed random parameters of real-time latency (https://wondernetwork.com/pings, accessed on 22 February 2021) for each of the different network links in the system.

The Kademlia implementation (http://peersim.sourceforge.net/, accessed on 19 December 2020) of PeerSim simulator [43] has been used for the performance experiments. The implementation has been modified to fit RDHT. In our implementation and as with uTorrent (https://www.utorrent.com/, accessed on 19 December 2020), the popular implementation of Kademlia, system wide replication is set to 8 and the lookup parallelism is set to 4. The results of researches [44,45] that focus on studying these two factors and other parameters in Kademlia [17] implementation to improve the lookup latency in DHT based implementation can be applied on RDHT.

**Figure 6:**
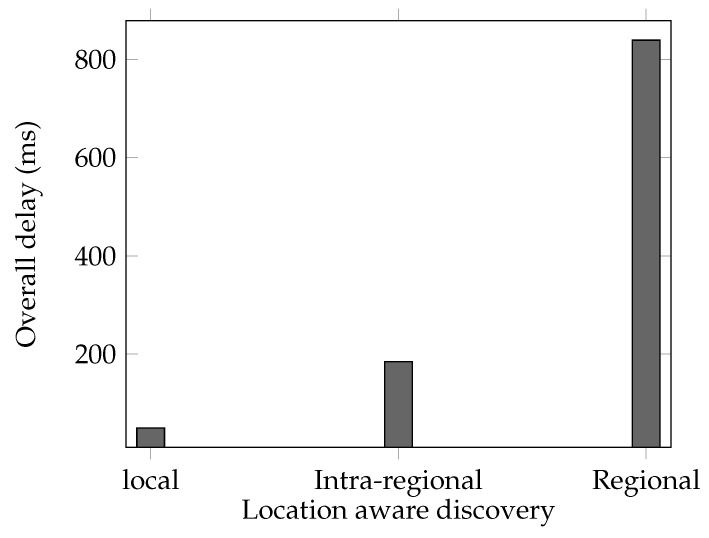
Regional resource registration delay in Attred.

The system performance has been tested using a simulated network with 400 million to 2 billion IoT gateways. The IoT gateways are distributed and grouped in 200 region sets with 200 regions per region set (i.e., overall 40,000 regions with 10,000 to 50,000 IoT gateways per region). Figure 6 shows the resource discovery latency in a local discovery (i.e., two nodes in the same local region), intra-regional discovery (i.e., two nodes in two different regions that are within the same region set) and regional discovery (i.e., two nodes in two regions that are in two different region sets). We assumed that no churn occurred and no cache has been used in Attred during the test.

The system performance has been tested using a simulated network with 2 billion IoT gateways. The IoT gateways are distributed and grouped in 200 region sets with 200 regions per region set (i.e., overall 40,000 regions with 50,000 IoT gateways per region). Figure 6 shows the resource discovery latency in a local discovery (i.e., two nodes in the same local region), intra-regional discovery (i.e., two nodes in two different regions that are within the same region set) and regional discovery (i.e., two nodes in two regions that are in two different region sets). We assumed that no churn occurred and no cache has been used in Attred during the test.

**Figure 7:**
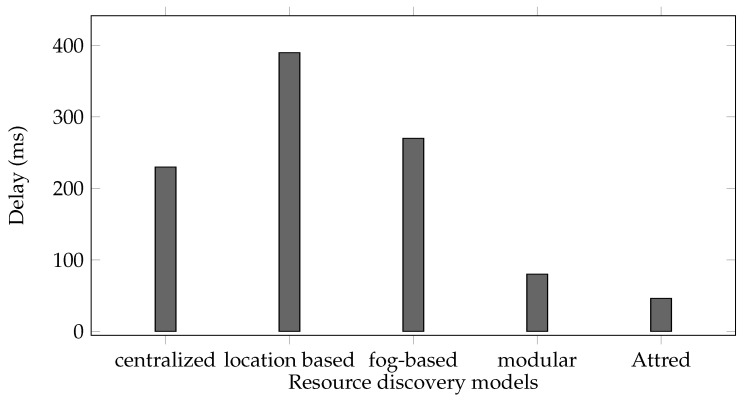
Resource discovery delay in different models.

As part of the evaluation and to study the efficiency of Attred, it has been compared with the centralized service discovery [3], location based distributed discovery [7], fog based distributed discovery [9] and modular discovery [20]. The direct matching scheme that has the minimum response time in the centralized model [3] has been used. The specifications of each of the models has been listed in Table 3 and the resource discovery delay has been illustrated in Figure 7. Evaluation shows that Attred meets the latency requirements in resource discovery for IoT when it is compared to different resource discovery models.

**Figure 8:**
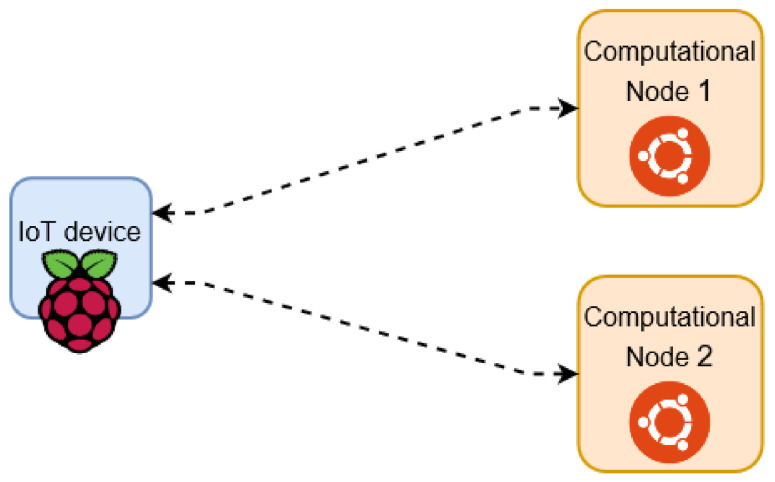
Performance evaluation of computation distribution in Attred.

It is feasible to implement and adopt ABE in number of IoT devices [46], but due to its high computation power, it takes a considerable time to perform the ABE operations in those resource constrained devices. The effectiveness of the proposed distributed computation in Attred was evaluated with a hardware setup consisting of a Raspberry Pi Zero ARM11 running at 1 GHz as the IoT device, and computers with 64-bit computing architecture and Intel Corei7 CPU running at 1.8 GHz with Ubuntu 20.04 as the computational nodes. Figure 8 illustrates the network configuration. The model was implemented in Python. Based on a note in [38], to improve the implementation efficiency prime order group was used.

In the first case of this scenario, the IoT device performed the encryption locally, and in the second case it used two computational nodes to distribute the computation workload of encryption during resource registration between those two nodes. During the test of our proposed model the communication delay was ignored and only the differences in the computations were measured. As it is shown in Table 4, the number of attributes in the discovery policy affect the required time. In addition, it is shown that the computation time in case of distributing the computations to the two computational nodes were improved by 77.3% comparing to perform them locally, with a five attribute discovery policy.

## 6. Conclusions

In this paper an attribute based resource discovery model for IoT (Attred) has been proposed. It adopts the peer to peer (P2P) scheme by utilizing Region-based Distributed Hash Table (RDHT), a proposed location-aware version of DHT. Attred ensures that there is no single point of failure in the system and the network can be easily scaled without any need of a reorganizing and synchronizing authority. The RDHT overlay is generated by taking into consideration the physical locations of IoT gateways in the system. Attred utilizes the decentralized ABE, which allows each organization to establish its own attributes authorities. The resources in Attred are registered based on some attributes that describe their properties. The set of attributes is not fixed, and new attributes can be added to the system in real-time. In addition, the resources set the attributes of the clients that are able to discover them in Attred through defining a discovery policy. The clients are able to discover the registered resources using one or more attributes of the required resources. Only those clients that have the required defined attributes in the discovery policy can discover the resources.

The used DABE during registration requires heavy computation. The distribution of heavy computations during resource registration allows the peers in RDHT to take the advantage of more powerful dedicated nodes such as cloud servers during the registration. This distribution improved the registration process without revealing the address data of the resources to those nodes or requiring real-time cooperation with them. The analysis results showed that Attred works efficiently and can provide the required security properties of discovery correctness, soundness, resource privacy and client privacy.

Some open problems remain related to Attred. The resources in the current model define the required attributes by clients to be able to discover them. Updating these attributes requires deleting the stored tuples and performing another registration of the modified tuples. This process in Attred needs to be modified in the future to allow the resources to cryptographically update the attributes in the overlay without a need to delete and replace them. The privacy of the client depends on either not including them in the discovery request or the assumption that the rainbow attack is hard. This privacy property and the assumptions can be improved in future works.

## Figures and Tables

**Table 1:** Supported properties in resource discovery models.

Features	Decentralized	Location Aware Overlay	Multi Attributes	Security Considerations	Controlled Discovery
Jara et al. [2]	✗	✗	✓	✗	✗
Jia et al. [3]	✗	✗	✓	✗	✗
Cheshire et al. [4]	✗	✗	✓	✓	✗
Mokadem et al. [5]	✓	✗	✗	✗	✗
Paganelli et al. [6]	✓	✗	✓	✗	✗
Cirani et al. [7]	✓	✓	✗	✗	✗
Tanganelli et al. [9]	✓	✗	✓	✗	✗
Cabrera et al. [11]	✓	✓	✓	✗	✗
Kamel et al. [8]	✓	✗	✓	✓	✗
Pahl et al. [20]	✓	✗	✓	✓	✓
Trabelsi et al. [23]	✓	✗	✓	✓	✓
Kamel et al. [10]	✓	✗	✓	✓	✓
Wang et al. [24]	✗	✗	✓	✓	✓
Attred	✓	✓	✓	✓	✓

**Table 2:** Network parameters.

Type	Parameter
local connection latency	2 ms
sub-regional latency (local region)	3–8 ms
intra-regional latency (region set)	10–30 ms
long distance latency	80–120 ms

**Table 3:** Resource discovery models.

Model	Approach	Properties
Jia et al. [3]	Centralized Resource Discovery	direct matching
Cirani et al. [7]	Location based discovery	5 hops
Tanganelli et al. [9]	Fog based discovery	100 nodes
Pahl et al. [20]	Modular discovery	4 predicates/search providers
Attred	Region discovery	10,000 nodes

**Table 4:** Effect of distributing the workload among computational nodes.

Number of Attributes	Local Execution Time (ms)	Distributed Execution Time (ms)		Improvement (%)
		IoT Gateway	Computational Nodes	
1	280	94	5	64.6
2	403	128	11	65.5
3	599	139	19	73.6
4	710	154	25	74.8
5	898	171	33	77.3

## Abbreviations

The following abbreviations are used in this manuscript:

AA	Attribute Authority
ABE	Attributed Based Encryption
CP	Cipher Policy
DABE	Decentralized Attributed Based Encryption
DHT	Distributed Hash Table
KP	Key Policy
LSSS	Linear Secret Sharing Scheme
MA	Multi Authority
RDHT	Region-Based Distributed Hash Table
